# Tear Down the Fluorescent Curtain: A New Fluorescence Suppression Method for Raman Microspectroscopic Analyses

**DOI:** 10.1038/s41598-019-52321-3

**Published:** 2019-10-31

**Authors:** Elena Yakubovskaya, Tatiana Zaliznyak, Joaquin Martínez Martínez, Gordon T. Taylor

**Affiliations:** 1 School Marine and Atmospheric Sciences, Stony Brook University, Stony Brook, NY 11794 USA; 2 Bigelow Laboratory for Ocean Sciences, East Boothbay, Maine, 04544 USA

**Keywords:** Molecular modelling, Cellular microbiology, Molecular imaging, Marine biology

## Abstract

The near exponential proliferation of published Raman microspectroscopic applications over the last decade bears witness to the strengths and versatility of this technology. However, laser-induced fluorescence often severely impedes its application to biological samples. Here we report a new approach for near complete elimination of laser-induced background fluorescence in highly pigmented biological specimens (e.g., microalgae) enabling interrogation by Raman microspectroscopy. Our simple chemiphotobleaching method combines mild hydrogen peroxide oxidation with broad spectrum visible light irradiation of the entire specimen. This treatment permits observing intracellular distributions of macromolecular pools, isotopic tracers, and even viral propagation within cells previously not amenable to Raman microspectroscopic examination. Our approach demonstrates the potential for confocal Raman microspectroscopy becoming an indispensable tool to obtain spatially-resolved data on the chemical composition of highly fluorescent biological samples from individual cells to environmental samples.

## Introduction

Raman microspectroscopy is an excellent tool to produce molecular fingerprints of biological specimens. This form of vibrational spectroscopy relies on inelastic scattering of monochromatic light to provide specific chemical information based on fundamental molecular vibrations. Aqueous samples are amenable to Raman spectroscopic analysis, making it a versatile tool capable of yielding a wealth of information on the identity and quantity of chemical species. Strengths of Raman spectroscopy include: high spatial resolution (<1 μm for micro-Raman setups), high spectral resolution, simple sample preparation procedures, high chemical specificity, and the possibility of label-free *in vivo* chemical mapping, all of which are particularly useful for biological, ecological, and biomedical applications^1^. Information about cell-to-cell and intracellular variations in metabolite concentrations or key biological macromolecules is critical to deeper mechanistic understandings in such diverse fields as cancer stem cell studies^2^, embryogenesis^3^, antibacterial drug development^4^, “fluxomics” (i.e., study of cellular metabolite fluxes)^5^, and environmental microbiology^6^. However, most conventional assays available to researchers are only capable of measuring ensemble-averaged concentrations, a fact which substantially limits progress in many lines of biological inquiry. Raman microspectroscopy is arguably one of the few methods that can fill this gap.

The near exponential proliferation of published Raman spectroscopic applications over the last decade bears witness to the strengths and versatility of this technology. Unfortunately, one serious challenge frequently curtailing Raman microspectroscopic probing of biological samples is laser-induced fluorescence. Autofluorescence background can be produced by tryptophan and tyrosine side chains in folded proteins, by carotenoids and photosynthetic pigments or other chromophores within microbial, animal and plant cells^7^. In most cases, radiation emitted by cellular autofluorescence is several orders of magnitude more intense than the Raman signal. Furthermore, spectrally-broad fluorescence emissions completely overlap with Raman-scattered radiation, and therefore cannot be separated by simple optical filters^8^. Consequently, analysis of pigmented biological samples is often limited to Resonance Raman spectroscopy (RRS) which selectively excites chromophores within their absorption bands in the visible region of the spectrum. Although RRS increases Raman scattering efficiency by several orders of magnitude, chemical compositional information of pigmented samples is generally limited to conjugated chains, such as carotenoids. Thus, to recognize additional underlying Raman vibrational modes and extract true peak intensities, fluorescence must be circumvented, suppressed or eliminated.

Several approaches have been used to solve this problem. With few exceptions, most fluorescence avoidance methods capitalize on differences in optical behavior of Raman and fluorescence excitation and emission radiation (e.g., lifetime, wavelength, line shape, polarization)^8^. Due to significantly longer fluorescence emission lifetime, the Raman signal can be extracted using various temporal domain methods^9^ or ultrafast pulsed laser and gated detection techniques^10^. Wavelength domain methods, such as excitation with a deep ultraviolet laser (UV-RRS) can produce nearly fluorescence-free spectra, but deliver potentially destructive radiation to delicate biological samples and require instruments totally outfitted with UV-transparent optics and UV-sensitive detectors^11^. Differences in Raman line shapes and the spectrally broad background fluorescence emissions allow extraction of the former using baseline correction algorithms^12^. Although these approaches are capable of circumventing a significant fraction of a sample’s fluorescence, most of them require special instrumentation, sophisticated data analysis techniques and can have medium to low data capture efficiency^8^.

Efficient detection of Raman signal in samples with strong autofluorescence can be attained using surface-enhanced Raman scattering (SERS) which is produced by analytes adsorbed to gold or silver surfaces^13^. The primary effect that makes detection possible is the dramatic increase in the Raman scattering cross-section of an analyte at the metal’s contact point (SERS enhancement factors can be as much as 10^14^)^14^. However, observation of intact biological systems by SERS requires use of metal nanoparticles^15^. So applicability of SERS is constrained by the ability to efficiently introduce and homogenously disperse gold or silver nanoparticles within cells. This is feasible for homogeneous liquid or thin film samples, but very challenging for intact cells or tissues. Alternatively, photobleaching of specific fluorophores (e.g., Cy3 fluorochrome) in specimens has been achieved by laser irradiation before Raman spectral acquisition^16^. However, this approach is time-consuming because each target spot requires 3–300 minutes of photobleaching prior to data acquisition, depending on the photostability of the fluorophore, which can vary considerably among fluorophores.

In summary, Raman spectral analyses within individual cells or tissues could be significantly improved if fluorescence were eliminated at the sample preparation stage. Such a method could then be used with conventional Raman microspectroscopic measurements and obviate the need for exotic instrumentation or complicated data analytical routines.

In this report, we present a new and simple chemiphotobleaching method to irreversibly suppress background fluorescence of biological specimens during sample preparation for Raman microspectroscopic analysis. We show that >99% of the entire sample’s background fluorescence is eliminated after simultaneous exposure to a low concentration of hydrogen peroxide (3%) and irradiation by a standard photodiode lamp for 0.5–2 hours. The level of background fluorescence of biological materials is sample-dependent. For each new sample type, the chemiphotobleaching time should be empirically optimized. In some highly recalcitrant fluorescent samples or in cases of limited sample availability which may preclude optimizing treatment time, we advocate chemiphotobleaching for 10 h. In rare cases where samples have residual fluorescence, the entire cell is subjected to brief (1–8 min) laser photobleaching on the Raman microspectroscope stage to totally quench fluorescence. Our approach is significantly less harmful to biological specimens than previously published chemical bleaching methods^17^ and requires no modification of standard Raman microspectrophotometers.

In our microbial ecological studies, we have applied this protocol to a variety of photosynthetic microorganisms (e.g., cyanobacteria, chlorophytes, prymnesiophytes, and bacillariophytes) which pose the most significant challenge to Raman interrogation because of their abundant autofluorescing pigments (chlorophylls, carotenoids, phycoerythrin, and phycobilin). Our protocol enabled direct detection of Raman peaks characteristic of nucleic acids, proteins, lipids and polysaccharides in all these organisms. Chemiphotobleaching treatment times were initially optimized with different types of preserved photosynthetic cells that possess strong autofluorescence (e.g., *Emiliania huxleyi and Tetraselmis levis)*, and then routinely used for all experiments with those microalgae. Efficient suppression of autofluorescence was consistently observed among at least 50 cells of each species. Analyzing an individual microalgal sample 1, 24, and 120 hours after completing chemiphotobleaching demonstrated that our method irreversibly quenches autofluorescence. Thus, intracellular macromolecular pools of formerly fluorescent cells can be routinely surveyed in stored post-treatment samples.

**Figure 1:**
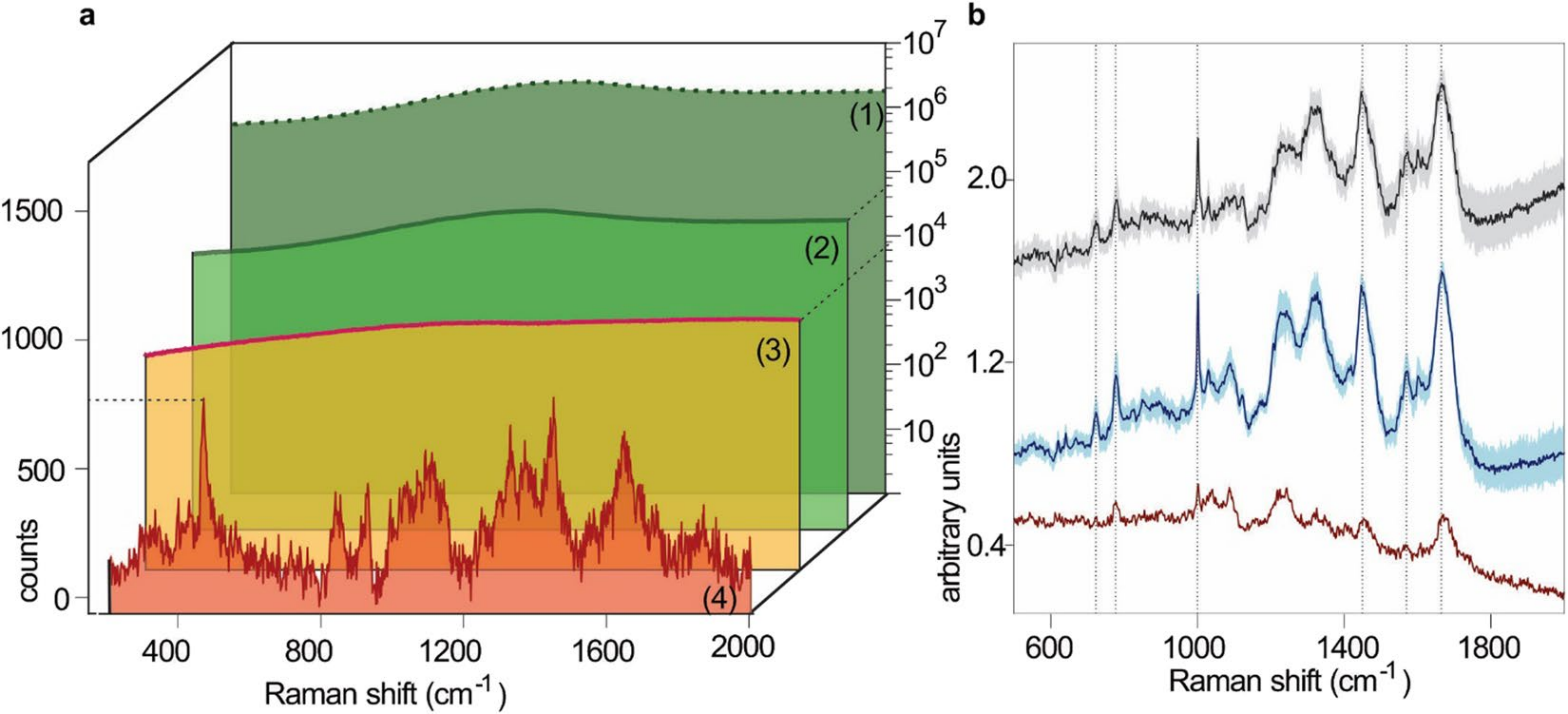
Chemiphotobleaching effectively suppresses background fluorescence encountered in Raman microspectroscopy without detectable cell damage. (**a**) Comparison of the efficiency of autofluorescence suppression using 633 nm laser photobleaching^1,2^ and chemiphotobleaching^3,4^. Estimated background fluorescence of an untreated, preserved *Tetraselmis levis* cell^1^ and measured after 500 seconds of photobleaching^2^. Signal from *T. levis* cell after chemiphotobleaching entire sample for 3 hours^3^. Raman spectrum obtained from chemiphotobleached *T. levis* cell after 500 sec of photobleaching entire cell^4^. Photobleaching of the whole cell was made under 20X objective lens at about 8 mW laser power at the sample (spectra 1–3). Spectrum^4^ was taken at a single point in the same *T. levis* cell, under 100X objective lens (NA = 0.9) in StreamHR scan mode with 4 mW laser power at the sample and 30 s exposure time. (**b**) Raman spectra of exponentially growing *Escherichia coli* bacterial cells lacking endogenous autofluorescing pigments. Cells were excited at 633 nm without (grey = control) and with (blue) 24 hours of chemiphotobleaching. Bottom spectrum is the control subtracted from the chemiphotobleached treatment, illustrating that no information is lost by chemiphotobleaching and that this treatment has slightly better signal to noise quality. Mean spectral intensities + 1 S.D. (n = 30 cells) are presented at each wavenumber (cm^−1^) as the solid lines and shaded areas, respectively.

The effect of chemiphotobleaching on the highly pigmented microalga *Tetraselmis levis* (a chlorophyte) is illustrated in Fig. 1a. Untreated cells exhibit strong background fluorescence that immediately saturates the detector. The approximated fluorescent signal (see methods) for an untreated cell exceeds 10^6^ counts across the entire spectral range during a 1 s detector exposure (Fig. 1a - trace 1). Unsaturated signal can only be collected from a single spot of an untreated cell after 500 seconds of photobleaching with 633 nm laser exposure (Fig. 1a - trace 2), but the cell still produces a very high level (10^5^ counts s^−1^) of broad background fluorescence. Prolonged (6 hours) spot-focused radiation with the laser beam, can reduce fluorescence level to 10^2^ counts s^−1^, but still does not yield a detailed Raman spectrum from an untreated *T. levis* cell. The initial spectrum of chemiphotobleached preserved cells exhibits some residual fluorescence at the level of 10^4^ counts s^−1^ (Fig. 1a - trace 3), but it decays after 500 seconds of photobleaching the entire cell with a 633 nm laser at low magnification and permits acquisition of a detailed Raman spectrum in 30 s (Fig. 1a - trace 4). This protocol obviates the need for spot-by-spot photobleaching and enables uninterrupted Raman interrogation of any part of a cell or mapping the whole cell of this formerly highly fluorescent specimen.

It is important to note that this procedure causes no alteration in chemical composition that is detectable by Raman spectroscopy. To illustrate, Raman spectra of bacteria (*E. coli)* cells preserved in 2% borate-buffered formaldehyde during exponential growth phase were recorded before (Fig. 1b; upper spectrum) and after the chemiphotobleaching procedure taken to an extreme (24 hr exposure) (averaged single-cell spectra; n = 30 cells) (Fig. 1b; middle spectrum). Even after this very prolonged treatment (10–20 times longer than required for efficient fluorescence suppression), no appreciable loss of information is evident in Raman spectra from treated cells as demonstrated by a difference spectrum - chemiphotobleached spectrum minus untreated spectrum (Fig. 1b; bottom spectrum). If anything, this analysis suggests that Raman spectra from preserved and chemiphotobleached cells have superior signal-to-noise characteristics.

To the best of our knowledge, this is the first report of fluorescence elimination in biological specimens by simultaneous exposure to hydrogen peroxide and broad spectrum light. The exact chemical mechanism of fluorescence suppression occurring during this procedure is not completely clear. It can be understood partly based on experiments that efficiently bleached fluorescent metal complexes^18^ or organic dyes^19–21^ subjected to H_2_O_2_/visible light treatments. In those studies, the proposed mechanisms included either direct activation of hydrogen peroxide by the excited fluorophore molecule^18^, or Fe^3+^ mediated generation of reactive oxygen species from H_2_O_2_ in the presence of the dye (“photo-Fenton oxidation”^19–21^). Based on these studies, we hypothesize that in our case fluorescence quenching involves two steps: light-induced transition of fluorophore molecules to the excited singlet state, and the interaction of the excited molecules with H_2_O_2_. In this process, cellular fluorophores that efficiently absorb light in the visible spectral region, transfer the absorbed light energy to hydrogen peroxide, thereby serving as photosensitizers. The fluorophore’s destruction occurs either directly, during the transfer process (similar to what was reported for iron oxalate complexes^18^), or as a result of attack on the fluorophore molecule by hydroxyl radicals generated from energy transfer to H_2_O_2_. The hydroxyl radicals generated further attack the fluorophores. In both cases, the fluorophore molecule is rapidly altered.

It is noteworthy that organic fluorophores are extremely efficient photosensitizers due to their high quantum absorption efficiencies, which dramatically amplify their photooxidation by H_2_O_2_. This creates a negative feedback loop, such that production of reactive oxygen species essentially halts when all fluorophore molecules have been bleached. This feedback minimizes oxidative destruction of non-fluorophores in the sample and reduces possible artifacts.

**Figure 2:**
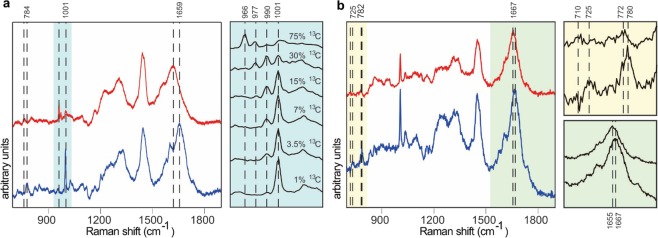
Application of the chemiphotobleaching protocol to Raman interrogation of cells from stable isotope probing (SIP) experiments. (**a**) Red shift of phenylalanine Raman peak in single-cell Raman spectra of chemiphotobleached *Emiliania huxleyi* (prymnesiophyte) grown with natural ^13^C abundances (blue spectrum) or augmented with 20 mM ^13^C –bicarbonate (red spectrum). Light blue panel shows phenylalanine Raman peak region (966–1001 cm^−1^) for preserved *E. huxleyi* cells grown with 0, 1.25, 2.5, 5, 10 or 20 mM ^13^C –bicarbonate. Propagation of phenylalanine peaks to left of the 1001 cm^−1^ peak indicate that cellular phenylalanine molecules are becoming isotopically heavy from 1% (natural abundance) to 3.5, 7, 15, 30 and 75% in the ascending spectra (red shift to 966 cm^−1^ for fully ^13^C-phe vs 1001 cm^−1^ for natural abundance). (**b**) Single-cell Raman spectra of the chemiphotobleached freshwater green alga, *Oophila amblystomatis*, grown in media with 100% ^15^NO_3_^−^ (red spectrum) compared to cells grown with same concentration of natural isotopic abundance NO_3_ (blue spectrum). Assimilation of ^15^NO_3_^−^ into macromolecules of *O. amblystomatis* can be detected by red-shifts in several spectral regions attributable to DNA/RNA base ring breathing mode from 725 to 710 cm^−1^ for adenine and from 784 to 772 cm^−1^ for cytosine/uracil (yellow panel). Amide I peak position also shifts from 1667 to 1655 cm^−1^ (green panel).

From the above, we can conclude that our chemiphotobleaching method allows collection of high quality Raman spectra from samples that are generally considered totally unsuitable for study using conventional Raman techniques. This is an enabling technology for new applications of Raman spectroscopy to biological specimens. Applications such as combining stable isotope probing (SIP) with confocal Raman microspectroscopy to analyze the molecular and isotopic composition of single cells, or following material fluxes from prey to predators or from a host to its viruses are now expedited. SIP is a means to measure assimilation of ^13^C or ^15^N -labelled substrates, whereby the presence of heavy isotope label can be easily detected as red-shifted Raman peaks (Fig. 2a,b).

To date, SIP measurements of samples with strong autofluorescence have been performed using a resonance Raman (RR) technique, whereby the laser frequency is close to the electronic transition energy band of the chromophore of interest, so that Raman scattering efficiency is enhanced by factors of 10^3^–10^5^ ^22^. Chromophores suitable for RR are characterized by strong absorbance in the visible spectral range. For example, endogenous carotenoids in photoautotrophic cells have been demonstrated to be excellent RR beacons for SIP measurements of activity and growth^23,24^. Unfortunately, this approach cannot be universally applied to all cells, tissues or organisms because carotenoids are sparce or totally absent in many organisms and can vary in composition among organisms that have them. Importantly, monitoring stable isotope assimilation relies on analysis of carotenoid peaks only and is strictly linked to their biosynthetic pathways which may or may not be closely synchronized with other biosynthetic pathways. In addition, RR measurements can only be used when the chromophore’s electronic transition energy band is close to a standard laser frequency. Otherwise, more exotic instruments with a tunable laser source are required.

SIP measurements using spontaneous Raman scattering by omnipresent aromatic groups, such as in phenylalanine (amino acid), are good alternatives to the carotenoid approach. However, the infamous “fluorescent curtain” often prohibits routine implementation of this approach. We demonstrate the possibility of SIP measurements in carotenoid and chlorophyll-bearing cells by combining our chemiphotobleaching procedure and conventional Raman scattering. The accumulation of red-shifted Raman peaks of phenylalanine’s phenyl ring in individual cells of the microalga, *E. huxleyi*, (a prymnesiophyte) grown in varying concentrations of ^13^C labelled bicarbonate is shown in Fig. 2a. With an appropriate calibration curve, the fractional abundance of ^13^C within individual cells and their organelles, as well as protein synthesis rates all can be calculated from such spectral data^16^.

Similarly, assimilation of ^15^N into other macromolecular pools can also be quantified in highly fluorescent cells after being subjected to our chemiphotobleaching protocol. For example, several Raman peaks are demonstrably red-shifted in cells of the freshwater green alga, *Oophila amblystomatis*, grown with 100% ^15^N-nitrate compared to cells grown with natural isotopic abundance nitrate (Fig. 2b). Raman peaks corresponding to DNA/RNA base ring breathing modes show clear shifts in Raman wavenumbers, e.g., from 725 to 710 cm^−1^ for adenine and from 784 to 772 cm^−1^ for cytosine/uracil. The amide I peak position also shifts from 1667 to 1655 cm^−1^ in cells assimilating ^15^N-nitrate. This shift may be a secondary effect because the amide I peak is primarily attributed to a C=O stretching vibration which does not involve N^25^.

**Figure 3:**
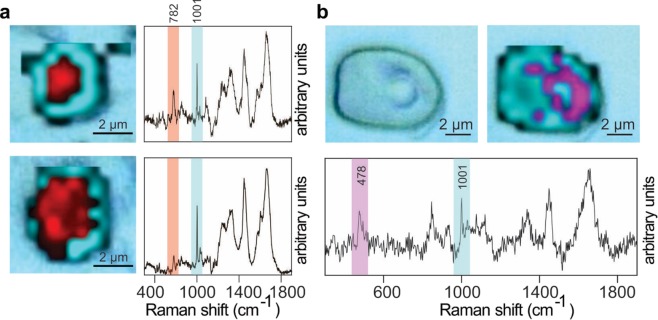
Label-free applications of the chemiphotobleaching protocol for single-cell Raman microspectroscopic chemical mapping. (**a**) Comparison of single-cell Raman microspectroscopic chemical maps of an uninfected preserved microalgal cell (*E. huxleyi*) (upper panel) and a cell from the same culture 5 days post-infection with the EhV-163 virus (lower panel) after chemiphotobleaching. Raman spectra from single spots showing relative intensities of diagnostic peaks for nucleic acids (784 cm^−1^) and protein (phenylalanine at 1001 cm^−1^) used to produce 2-D chemical maps are shown to the right. (**b**) A reflected bright-field image of the microalga, *T. levis* cell (left) and single-cell Raman microspectroscopic chemical map of the same cell after chemiphotobleaching protocol (right). Accumulation of starch granules surrounding the pyrenoid body as culture approached phosphorus limitation was monitored by intensities and spatial distributions of Raman scattered emissions from polymeric glucose (purple) and superimposed on the cellular protein distribution (blue). Raman spectrum from single spot showing diagnostic peaks for starch (478 cm^−1^) and protein (phenylalanine at 1001 cm^−1^) used to produce 2-D maps is presented below map.

Efficient single-cell Raman mapping is another application enabled once background fluorescence is eliminated from the entire specimen. The possibility of precise detection of characteristic spectral features of major cellular pools, such as amino acid or nucleotide residues, uniquely allows spatial resolution of variations in chemical composition within an individual cell. For example, single-cell Raman microspectroscopic chemical maps were produced from an uninfected preserved microalgal cell (*Emiliania huxleyi*) (upper image; Fig. 3a) and from a cell infected with the EhV-163 virus (lower image; Fig. 3a) In these images, phenylalanine aromatic and guanine 2′-endo sugar peak intensities are represented as pseudo-colors, blue and red, respectively. Propagation of nascent viral particles is clearly seen by comparing the single, compact nuclear DNA-rich domain in the healthy cell with the multiple, dispersed DNA-rich domains in the infected host’s cytoplasm.

Raman microspectroscopic chemical maps can also be used to assess nutritional status of individual cells by measuring storage products, such as polyphosphates or polyhydroxyalkanoates^26,27^, but this has been challenging in pigmented cells. Figure 3b demonstrates that intracellular distributions of polymeric glucose (starch) in the microalga, *T. levis*, can be mapped by Raman microspectroscopy once the entire preserved specimen is subjected to chemiphotobleaching. Intensities of Raman scattered emissions from starch and phenylalanine (a protein proxy) are mapped as purple and blue, respectively. This image illustrates accumulation of starch granules surrounding the cell’s pyrenoid body as the culture approached phosphorus limitation. These examples of direct label-free intracellular visualization of viral propagation and polysaccharide accumulation, which are challenging to visualize by other techniques, demonstrate some of the capabilities of our approach and its potentially wide range of applications.

**Figure 4:**
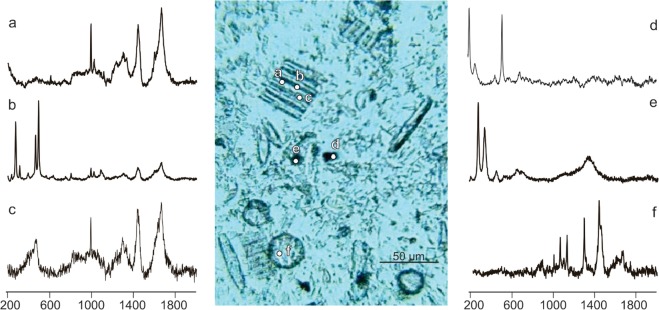
Raman spectra of Arctic spring bloom material captured in an oceanographic sediment trap deployed in 30 meters of water. Post recovery, these highly autofluorescent samples were filtered and frozen until laboratory chemiphotobleaching procedure for 24 hours in hydrogen peroxide, 7 hours of which were illuminated, see methods). Spectra *a-c* were obtained from different locations in a single chain of pennate diatoms and illustrate presence of typical cytoplasm (**a**) and what appear to be signatures of albite (NaAlSi_3_O_8_) (**b**) and analcime (NaAlSi_2_O_6_*H_2_O) (**c**) associated with the frustules. Spectrum *d* from a 4 × 6 μm opaque particle indicates a quartz (SiO_2_) and anatase (TiO_2_) composition. Target *e*, similar in appearance to target *d* is clearly composed of hematite (Fe_2_O_3_), a common form of iron oxide. Target *f* is in a *Thalassiosira* diatom cell and it yields a spectrum indicative of proteins, nucleic acids, as well as characteristic peaks for saturated carbon chains, distinctive for fatty acids.

Confocal Raman microspectroscopy offers an approach to examine microspatial distributions of a wide range of analytes within and among unicellular organisms and particles in environmental samples. For example, we surveyed material from an oceanographic sediment trap which captured biota and particles sinking through the ocean’s upper 30 meters during an Arctic spring bloom. This preserved material is dominated by heavily pigmented microalgae (primarily diatoms) and biogenic debris which would be extremely impractical, if not impossible, to scan without our chemiphotobleaching pre-treatment. Analysis of a single pennate diatom chain (Fig. 4, targets *a-c*) illustrates how spatial heterogeneity in chemistry within and between cells can be elucidated. In this fluorescence-tamed sample, spectral information can be extracted from the cells’ cytoplasm and silica shell (frustule), such as inclusion of the minerals albite and analcime. The cytoplasmic fingerprint (spectrum *a*) in this pennate chain is qualitatively similar to the dominant *Thalassiosira* observed in these samples (target *f*). Both the pennate chain and *Thalassiosira* cell share cytoplasmic signatures for proteins (1001, 1446, and 1671 cm^−1^) and nucleic acids (765 cm^−1^), but target *f* is clearly enriched in fatty acids (1061, 1127, 1294, and 1438 cm^−1^) compared to target *a*. Captured particles which were not visually recognizable (Fig. 4, targets *d, e*) yielded Raman spectra indicative of quartz-anatase and hematite compositions, respectively. Despite the long chemiphotobleaching protocol, this challenging field sample still required brief laser photobleaching (20–60 s) over the region of interest to acquire fluorescence-free spectra. However, these observations establish that the chemiphotobleaching protocol effectively enables efficient Raman spectral acquisition from pigment-rich field samples without requiring prohibitively long laser photobleaching spot-by-spot.

## Conclusions

The above examples demonstrate the potential for confocal Raman microspectroscopy becoming an indispensable tool for obtaining spatially-resolved data on the chemical composition of highly fluorescent biological samples, including individual cells, tissues, and environmental samples. Concealment of the Raman signal by background fluorescence has been one of the major obstacles impeding wider application of this powerful technique to numerous subdisciplines, e.g., cell biology, metabolomics, “fluxomics”, environmental microbiology, and biogeochemistry. We anticipate that our chemiphotobleaching protocol will enable researchers to figuratively tear down “The Fluorescent Curtain” within challenging specimens and literally trace cellular assimilation of isotopic tracers, document intracellular biochemical changes, monitor intracellular viral propagation, and analyze very diverse field samples by Raman microspectroscopy.

## Methods

### Microalgal cultivation and viral infection

Cultures of the prymnesiophyte, *Emiliania huxleyi* (calcifying strain CCMP3266) and the chlorophyte, *Tetraselmis levis*, were grown in batch mode in sterile filtered (0.22 µm) seawater at 20 °C in f/2-Si medium^28^ on a rotating platform that assured uniform light exposure (48–63 μmol quanta m^−2^ s^−1^) during a 12:12 h light/dark cycle. The *E. huxleyi* culture was infected with the virus, EhV-163^29^, using a 1:100 volumetric ratio of viral lysate to culture. Samples of infected cells were taken every 12 hours for 7 days and then preserved with 2% borate-buffered formaldehyde (final concentration) and stored at 4 °C.

### Stable isotope probing (SIP)

*E. huxleyi* cultures were grown in f/2 media in which total dissolved inorganic carbon was augmented with varying proportions of ^13^C-bicarbonate. The ^12^C- and ^13^C-bicarbonate solutions (Cambridge Isotope Laboratories, Inc. Andover, MA; 99% ^13^C, 97% chemical purity) were prepared as 1 M working stocks. Nutrient and bicarbonate solutions were aseptically added directly to autoclaved 0.22 μm filtered seawater. Cultures were grown in 125 ml sterile non–vented PC Erlenmeyer flasks in f/2-Si media with an addition of ^12^C-bicarbonate solutions (control flasks) or ^13^C-bicarbonate solutions at following final concentration: 0, 1.25, 2.5, 5, 10 and 20 mM. Cultures were grown for 10 days and subsampled daily to determine growth rates, which did not significantly vary among treatments. Day 1 and Day 10 samples (3 ml) were fixed with 2% borate-buffered formaldehyde (final concentration) and stored at 4 °C until Raman spectroscopic analysis.

Preserved samples of the freshwater microalga, *Oophila amblystomatis*, were provided by Dr. John A. Burns (American Museum of Natural History, NY). Replicate cultures of this chlorophyte were grown in modified AF6 medium that was either provided with ^15^N-NaNO_3_ (98% ^15^N, Cambridge Isotope Laboratories, Inc.) or ^14^N-NaNO_3_ as their sole source of fixed nitrogen^30^. Samples withdrawn after ~6.5 doublings were preserved with 4% paraformaldehyde and captured on 0.2 μm GTTP membranes (EMD Millipore^®^) which were stored at −20 °C prior to processing for Raman interrogation.

### Marine debris sample collection

Material was collected from a 30-m surface-tethered oceanographic sediment trap during an Arctic spring diatom bloom (provided by Dr. Jeffrey W. Krause, Dauphin Island Sea Lab). The dominant diatom genus observed was *Thalassiosira*. Unpreserved trap water (0.7 L) was filtered onto a 1.2 µm polycarbonate membrane filter and immediately frozen at −20 °C in a petri dish. In the laboratory, a subsample of the filter was cut (~10% of the filter area) and chemiphotobleached in a petri dish as detailed below. After treatment, material was resuspended in particle-free seawater and filtered onto a 0.2 µm polycarbonate membrane filter, rinsed with deionized water, and freeze transferred as described below.

### Chemiphotobleaching protocol

Appropriate volumes of preserved phytoplankton cultures (1–3 ml) were passed through 0.2 μm GTTP membranes. Membranes were air-dried for 10 min on Whatman filter paper and placed in 1 inch Petri dish with 3% hydrogen peroxide (diluted from 30% in MilliQ H_2_O) under a bright white (3695 K), 251 lumens LED desk lamp (Hampton Bay^®^ #1000 052 866) for 0.5–7 hours. To ensure sufficient irradiance, light source was < 3 cm from the sample surface. For each cell type, time of chemiphotobleaching was optimized individually. (*E. huxleyi* – 1 h; *T. levis* – 3 h; O. *amblystomatis* – 2 h; marine debris – 7 h). Note – for marine debris, the light was applied for 7 of the 24 h peroxide exposure). After chemiphotobleaching, cells were freeze transferred to mirror-finished stainless steel slides suitable for Raman microspectroscopic analysis^31^.

### Raman measurements and spectral analysis

All Raman measurements were performed using a Renishaw® inVia™ confocal Raman microspectrophotometer configured with a modified upright Leica® DM2700™ fluorescence microscope and a computer-controlled motorized XYZ stage (0.1 μm step size). A He-Ne laser (633 nm wavelength) was used as the excitation light source. Spectra were acquired using a 1200 line/mm diffractive grating, centered at 1150 cm^−1^ and allowing 217–2050 cm^−1^ wavenumber coverage with 1.8 cm^−1^ spectral resolution and Raman-scattered emissions were imaged on a thermo-electric cooled 1024 × 256 CCD detector.

Sometimes brief laser bleaching was still required after chemiphotobleaching, but instead of hours of laser bleaching each spot, only several minutes at most were required to bleach an entire cell at low magnification. When required, the whole microscopic field was photobleached under a 20X objective lens at about 8 mW laser power at the sample for 500 s. Single point spectra of specimens were then chosen from a linear 4-point map acquired under a 100X objective lens (NA = 0.9) in StreamHR scan mode with 4 mW laser power at the sample and 30 s exposure times at each point.

Depending on the laser wavelength and objective lens used, spot sizes were in the order of 0.5–2 μm, so single point spectra integrate information from numerous components in the cytoplasm. If the size of the cell is similar to the laser spot size, then the spectrum obtained approximated an integral of the entire cell. Spectra from multiple cells were averaged at each wavenumber to get mean spectral intensities and peak shifts as well as sample-to-sample variability (S.D.).

Two-dimensional mapping of both viral-infected and uninfected *E. huxleyi* preserved cells was performed under a 100X objective lens in StreamHR scan mode. Laser power at the sample was about 4 mW and an exposure time of 30 s for each scan was used (110 scans for infected and 100 scans for control cell in total). Maps were acquired with a 0.5 μm step size in both x and y directions. Mapping of *T. levis* cells was done under the same conditions, except step size was 1μm in both directions and exposure time at each point was reduced to 15 s (130 scans in total).

In the *T. levis* study of fluorescence suppression (Fig. 1a), the initial fluorescence intensity of untreated cells cannot be directly measured due to signal saturation of CCD array. Therefore, to approximate the initial condition we calculated fluorescence decay induced by photobleaching as follows. Both untreated and chemiphotobleached cells were subjected to 50 photobleaching cycles of 10 s exposures to He-Ne laser (633 nm, 8 mW at sample) through a 20 x objective and 1 s spectra were recorded after each cycle. To derive total fluorescence loss from the chemiphotobleached cells, intensity ratios at each wavenumber of consecutive spectra were averaged over each of the 49 pairs of spectra, and the cumulative product yielded a final multiplication factor. On the assumption that the photobleaching effect is the same in untreated and treated cells, the initial fluorescence intensity of the former was approximated by applying the derived multiplication factor to the spectrum of the untreated cell after 50 photobleaching cycles.

Spectra were processed using Renishaw’s® Wire 4.1™ software, whereby each spectrum was subjected to baseline correction by fitting a 4^th^ order polynomial, followed by intensity normalization (range = 0–1). The quantitative analysis of ^13^C propagation in *E. huxleyi* cells was done by fitting the experimental data inside the 950–1050 cm^−1^ range to four separate full Voigt curve-fitting routines corresponding to four different Raman shifts of phenylalanine ring breathing modes with 0 (1001 cm^−1^), 2 (990 cm^−1^), 4 (977 cm^−1^), or all 6 (966 cm^−1^) heavy carbon atoms included in the ring. Based on the area of the fitted peaks, fractional abundance of heavy atoms among the total carbon was calculated.

## Data Availability

Data presented in this study is available upon request to the corresponding author.
